# Dietary resveratrol and β-Hydroxy-β-Methylbutyric acid enhance flavor and modulate intramuscular fat in Tibetan sheep: insights from transcriptomics and lipidomics

**DOI:** 10.3389/fvets.2025.1634086

**Published:** 2025-09-03

**Authors:** Xuan Chen, Qiurong Ji, Zhenling Wu, Fengshuo Zhang, Quyangangmao Su, Tingli He, Kaina Zhu, Shengzhen Hou, Linsheng Gui

**Affiliations:** College of Agriculture and Animal Husbandry, Qinghai University, Xining, China

**Keywords:** β-Hydroxy-β-Methylbutyric acid, transcriptomics, lipid metabolites, resveratrol, Tibetan sheep

## Abstract

**Introduction:**

This study investigated the effects of dietary resveratrol (RES) and β-Hydroxy β-Methylbutyrate (HMB) on immune function, oxidative status, and morphological changes in intermuscular fat of Tibetan sheep. Previous research suggests that RES and HMB may enhance muscle quality and lipid metabolism, but their combined effects on meat flavor, fatty acid composition, and underlying molecular mechanisms remain unclear. Therefore, we employed transcriptomics and lipid metabolomics to explore how RES and HMB synergistically regulate key signaling pathways and lipid metabolites to improve meat quality.

**Methods:**

A total of 120 male Tibetan lambs with similar initial body weight (15.5 ± 0.14 kg) were randomly divided into four groups (n = 30 per group): 1) H group (basal diet without RES or HMB); 2) H-RES group (1.5 g/day RES); 3) H-HMB group (1.25 g/day HMB); and 4) H-RES-HMB group (1.5 g/day RES + 1.25 g/day HMB). The experiment lasted 100 days, including a 10-day pre-test period and a 90-day formal trial. Intermuscular fat morphology, fatty acid composition, and flavor compounds were analyzed. Transcriptomic and lipid metabolomic approaches were used to identify differentially expressed genes and lipid metabolites, followed by pathway enrichment analysis to elucidate regulatory mechanisms.

**Results:**

The H-RES-HMB group exhibited significantly reduced intermuscular adipocyte area and diameter (*p* < 0.05) but increased cell density. Among medium- and long-chain fatty acids, the H-RES-HMB group showed significantly decreased SFAs (C17:0 and C18:0) (*p* < 0.05) and significantly increased MUFAs (C15:1N5 and C18:1N9) and PUFAs (C18:2N6, C18:3N6, C18:3N3, C20:3N6, and C20:3N3) (*p* < 0.05). Additionally, flavor compounds such as 2-Hexanone, 3-Hexanone, 3-Pentanone, and Methyl acetate were significantly elevated in the H-RES-HMB group (*p* < 0.05). Transcriptomic analysis revealed that RES and HMB synergistically regulated the Calcium (*ERBB4*, *P2RX7*, *ERBB3*, *P2RX3*, and *SLC8A1*), Hippo (*WNT9A*, *WNT10B*, *WNT6*, and *WNT2B*), Estrogen (*HSP90AA1*, *TGFA*, and *RARA*), and Arachidonic acid (*PLA2G4A*, *ALOX12*, and *PTGDS*) signaling pathways, collectively promoting muscle cell proliferation and differentiation, Metabolomics identified key lipid molecules (LPC(20:0/20:1), PC(21:2/37:0/38:5)) and pathways (Glycerophospholipid, Arachidonic acid metabolism) contributing to flavor optimization. Integrated analysis highlighted the *PLA2G4A*-AA-*ALOX12*/*PTGDS* axis as a central hub for flavor regulation.

**Discussion:**

The findings demonstrate that RES and HMB synergistically improve meat quality by modulating lipid metabolism and inflammatory responses. The reduction in SFAs and increase in MUFAs/PUFAs align with enhanced nutritional value, while elevated ketones/esters contribute to favorable flavor profiles. The transcriptomic and metabolomic integration reveals that *PLA2G4A* hydrolyzes PC(38:5) to release AA, which is metabolized via *ALOX12/PTGDS* to generate flavor precursors (generating 12-HPETE and PGD2). These mechanisms explain the “reduced off-flavor and enhanced aroma” effect. Future studies should validate these pathways in other livestock to assess broader applicability.

## 1 Introduction

In recent years, the large-scale and intensive development of animal husbandry in China, coupled with rising consumer demands for higher-quality livestock products, has led to a growing shortage of premium feed resources, posing a major constraint to industry growth (1). Nevertheless, China is rich in feed additive resources, which hold great potential for feed innovation from a nutritional standpoint (2). Resveratrol (RES), a natural polyphenolic compound (C₁₄H₁₂O₃), is widely distributed in plants such as grapes, peanuts, and *Polygonum cuspidatum*, and exhibits potent antioxidant, anti-inflammatory, and metabolic regulatory effects (3). Recent studies have demonstrated its efficacy in modulating lipid oxidation and improving oxidative stability of unsaturated fatty acids in food systems (4, 5). Studies have demonstrated that RES can upregulate the expression of adipogenesis-related genes, including PPARγ and C/EBPα, thereby promoting preadipocyte differentiation and lipid accumulation, which significantly enhances intramuscular fat (IMF) deposition (6). Additionally, RES influences desaturase enzymes such as SCD, improving the proportion of unsaturated fatty acids in muscle tissue and increasing the production of flavor precursors such as aldehydes and ketones, ultimately enhancing meat tenderness, juiciness, and flavor quality (7, 8). β-hydroxy-β-methylbutyrate (HMB), a key bioactive metabolite of leucine (C₅H₁₀O₃), exerts notable effects on protein synthesis and metabolic regulation in animals (9). Research has shown that HMB activates the mTOR signaling pathway to promote adipocyte differentiation and increases the expression of lipogenic enzymes such as ACC, effectively boosting IMF content (10, 11). Moreover, HMB suppresses the activity of lipolytic enzymes like HSL, thereby reducing lipid breakdown and maintaining optimal IMF levels, which contributes to improved marbling, tenderness, and flavor characteristics of meat (12, 13).

Adipose tissue is a metabolically active and heterogeneous endocrine organ (14). Studies suggest that IMF enhances meat quality through several mechanisms: firstly, its physical distribution separates muscle fiber bundles, reducing muscle density and improving tenderness (15). Secondly, during cooking, volatile compounds derived from IMF, including aldehydes and ketones, play a critical role in developing meat aroma (16). Additionally, melted fat acts as a lubricant for muscle fibers, enhancing juiciness (17). Importantly, IMF also modulates key lipogenic genes such as PPARγ, thereby influencing the formation of meat quality traits (18). Although regulating lipid metabolism is essential for improving meat quality, research on the effects of RES and HMB on IMF deposition and flavor precursor synthesis in Tibetan sheep remains limited. This study aims to investigate the regulatory roles of RES and HMB in IMF accumulation and the metabolism of flavor-related compounds in Tibetan sheep, providing a theoretical basis for the production of high-quality mutton.

Transcriptomic analysis (RNA-seq) enables the precise identification of key genes involved in adipogenesis (19), while lipidomics offers a comprehensive overview of lipid metabolic networks (20). The integration of transcriptomics and lipidomics not only clarifies gene-metabolite interactions but also uncovers the molecular mechanisms underlying IMF deposition, which directly impacts meat tenderness and flavor (21). Based on this rationale, the present study utilizes an integrated transcriptomic and lipidomic approach to systematically investigate the molecular basis of IMF accumulation in Tibetan sheep. This strategy aims to identify critical regulatory genes and characteristic lipid metabolites associated with fat deposition, thereby supporting efforts to enhance meat quality in Tibetan sheep.

## 2 Materials and methods

### 2.1 Animal feed and sample collection

For this experiment, 120 healthy 2-month-old Tibetan lambs with similar body weight (16.87 ± 0.31 kg) were randomly divided into 4 treatment groups (30 lambs per group), with 5 replicates per group (6 lambs per replicate). The four treatment groups were: basal diet group (H group), basal diet + resveratrol (1.5 g/day) group (H-RES group), basal diet + β-hydroxy-β-methylbutyrate (1.25 g/day) group (H-HMB group), and basal diet + resveratrol (1.5 g/day) + β-hydroxy-β-methylbutyrate (1.25 g/day) group (H-RES-HMB group). The experimental period lasted 100 days, consisting of a 10-day adaptation period followed by a 90-day formal trial. All lambs were housed in well-ventilated, dry pens with access to an exercise yard allowing free movement. The pens were thoroughly disinfected prior to the experiment. According to the experimental design, each treatment group received feed twice daily (08:00 and 17:00) (Table 1).

**Table 1:** Composition and nutrient level of the base ration (DM) %.

Item	Content
Corn	51.50
Soybean meal	2.00
Canola meal	12.80
Cottonseed meal	2.00
Palm meal	25.00
NaCl	1.00
Limestone	1.00
Baking soda	0.10
Premix^a^	4.60
Nutrient composition	
Digestible energy/(MJ kg^−1^)	12.71
Crude protein	14.27
Ether extract	3.29
Crude fiber	11.64
Neutral detergent fiber	26.70
Acid detergent fiber	19.97
Ca	0.84
P	0.40

a Provided per kilogram of diets: Cu 18 mg, Fe 66 mg, Zn 30 mg, Mn 48 mg, Se 0.36 mg, I 0.6 mg, Co 0.24 mg, VA 24,000 IU, VD 4,800 IU, VE 48 IU. Digestible energy is a calculated value, and the rest are measured values.

### 2.2 Determination and analysis of medium and long-chain fatty acid content

Following slaughter, adipose tissue samples were collected under aseptic conditions, immediately flash-frozen in liquid nitrogen, and stored at −80 °C until further analysis. Fatty acid analysis was performed using the hydrolysis-extraction method described in the national standard “Determination of Fatty Acids in Food” (GB 5009.168-2016). Briefly, an internal standard (undecanoic triglyceride solution) was added to each adipose sample, which was then hydrolyzed with hydrochloric acid. The liberated lipids were extracted into an ether phase, followed by saponification and methylation under alkaline conditions to produce fatty acid methyl esters (FAMEs). The resulting FAMEs were quantified using an Agilent GC-6890 gas chromatograph. Medium- and long-chain fatty acid contents were calculated based on the concentrations of individual FAMEs and their corresponding conversion factors.

### 2.3 Comparative analysis of volatile components in Tibetan mutton

A 1.0 mL sample of Tibetan sheep intermuscular fat was accurately weighed and placed in a 20 mL headspace vial, then incubated at 50 °C and 500 r/min for 20 min before injection. Each sample was analyzed in triplicate, with an injection volume of 500 μL (splitless mode, syringe temperature 85 °C). Gas chromatography analysis employed a column with an initial temperature of 40 °C, using high-purity nitrogen (≥99.999%) as the carrier gas. The pressure programming was as follows: initial flow rate of 2.0 mL/min held for 2 min, increased to 10.0 mL/min over 8 min, then raised to 100.0 mL/min over 10 min and held for 10 min (total run time 20 min, injection port temperature 80 °C). Ion mobility spectrometry utilized a tritium source for ionization (drift tube length 53 mm, electric field strength 500 V/cm, temperature 45 °C), with high-purity nitrogen (≥99.999%) as the drift gas at 150 mL/min in positive ion mode. The experiment first established calibration curves for retention time and retention index using a mixed standard solution of six ketones. The retention index of target compounds was calculated based on their retention times, followed by qualitative analysis using the NIST 2020 database and IMS drift time database in VOCal software. Volatile compounds were visualized using Reporter, Gallery Plot, and Dynamic PCA plugins to generate three-dimensional spectra, two-dimensional spectra, differential spectra, fingerprint plots, and PCA plots for comparative analysis of sample differences.

### 2.4 Histological analysis of intermuscular fat

Adipose tissue samples measuring approximately 3 × 3 cm were fixed in 4% paraformaldehyde for histopathological examination. Adipocyte volume was assessed using standard hematoxylin and eosin (H&E) staining. Briefly, the fixed tissue was sectioned into 5 μm-thick slices and subsequently stained with H&E. Adipose tissue morphology was observed under a light microscope at 40× magnification (Olympus DP2-BSW, Tokyo, Japan).

### 2.5 RNA-seq and data analysis

Total RNA was extracted from liver tissue using the TRIzol reagent kit (Invitrogen, Waltham, MA, United States), and RNA quality was assessed using an Agilent 2100 Bioanalyzer (Agilent Technologies, Santa Clara, CA, United States) and RNase-free agarose gel electrophoresis. PolyA mRNA was enriched using Oligo(dT) magnetic beads and fragmented via ultrasonication. The fragmented mRNA was used as a template with random oligonucleotides as primers to synthesize double-stranded cDNA using M-MuLV reverse transcriptase. After end repair, adapter ligation, and size selection with AMPure XP beads, a cDNA library (~200 bp) was constructed. Clean reads were aligned to the reference sequence using HISAT 2.2.4 software (22), and gene expression levels were quantified based on the FPKM method. DEGs were identified using DESeq2 under a False Discovery Rate (FDR) of 0.05. Genes with *p* < 0.05 and |log2FC| ≥2 were recognized as DEGs (23). Finally, GO and KEGG enrichment analyses were performed in R based on the hypergeometric distribution (*p* < 0.05 considered significant). To ensure sequencing quality, stringent criteria were applied for library quality assessment: RNA integrity and DNA contamination were evaluated by agarose gel electrophoresis, RNA purity and concentration were measured via spectrophotometry (OD260/280 and OD260/230 ratios), RNA concentration was quantified using a Qubit 2.0 Fluorometer (Thermo Fisher, Waltham, MA, United States), and RNA integrity was precisely determined using the 2100 Bioanalyzer (Agilent, CA, United States).

### 2.6 Validation of RNA-seq results with quantitative reverse transcription polymerase chain reaction

RNA samples were reverse-transcribed into complementary DNA (cDNA) using a reverse transcription kit (Takara, Dalian, China). The standard reverse transcription reaction mixture consisted of 1 μg total RNA, 1 μL random or Oligo(dT) primers, 4 μL 5 × reverse transcription buffer, 1 μL dNTP mix, 1 μL reverse transcriptase, 0.5 μL RNase inhibitor, and RNase-free H₂O to a final volume of 20 μL. GAPDH was used as the reference gene to normalize target gene expression levels. Quantitative polymerase chain reaction (qPCR) was performed using an Applied Biosystems 7500 Fast Real-Time PCR System (Applied Biosystems, United States) with the SYBR^®^ Premix Ex Taq^™^ Kit (TaKaRa, Dalian, China). The thermal cycling conditions were as follows: initial denaturation at 95 °C for 15 min, followed by 40 cycles of denaturation at 95 °C for 10 s and annealing at 60 °C for 30 s. The relative expression levels of target genes were calculated using the 2^−ΔΔCt^ method. Primer sequences are listed in Table 2.

**Table 2:** Primers used in qRT-PCR.

Name	Primer sequence (5′ to 3′)	Tm (°C)	Product length
NCBI: 101103889	TGGATGTGGGATCAGACCTCAGTG	60.0	96 bp
*ERBB3*	CTGGAGTTGTGCTGGCGTTGG		
NCBI: 101104779	TGCGACATCATCCTGCTGAACTTC	60.0	145 bp
*P2RX3*	ACTGCTTCTCCTCCGTGGTCTG		
NCBI: 101116716	ACCCAGGACCAGTATGCGGATG	60.0	110 bp
*SLC8A1*	GCGTGGTAGATGGCAGCGATG		
NCBI: 101102060	TGGTGGAGGCGGTCAGCATG	60.0	146 bp
*WNT9A*	GGAGGAGATGGCGTAAAGGAAAGC		
NCBI: 100127209	CGTCTGCTTCTGGTGATGAGATGG	60.0	115 bp
*HSP90AA1*	GAGTTGGCTACCTGGTCCTTTGTC		
NCBI: 101107973	GCTTCACCACCCTCACCATTGC	60.0	127 bp
*RARA*	AGCCCGTCCGAGAAGGTCATC		
NCBI: 101111472	AGAGAAGGACGTGCCGGGAAG	60.0	126 bp
*PLA2G4A*	GGCATCCAGTTCGTCTTCATCCAG		
NCBI: 101113685	ACACCAGGGCACGGACTCAG	60.0	135 bp
*ALOX12*	CAAGAGGACAGAGGGAGCGGTAG		
NCBI: 443192	GAAGAGGAAGGTGCTGTCCATGTG	60.0	81 bp
*PTGDS*	AGGAAGGTAGAGGTGACGTTGAGG		
NCBI: 101102299	GTCTCCTGTTCCTGGCGTTGTG	60.0	104 bp
*WNT10B*	GCAGACTGTGTTGGCGGTCAG		

### 2.7 Metabolite content determination

Prior to lipid analysis, frozen adipose tissue was ground into powder using a mortar and liquid nitrogen. Lipids from subcutaneous fat were extracted via tissue homogenization using an MTBE-methanol–water three-phase solvent system. Lipid profiling was performed on a UPLC-HRMS system (Thermo Scientific, Waltham, United States) equipped with a 50 °C Accucore C30 column, using mobile phases consisting of 60% acetonitrile aqueous solution (A) containing 10 mM ammonium formate and 0.1% formic acid, and 10% acetonitrile-isopropanol solution (B). Mass spectrometry detection was conducted in both positive and negative electrospray ionization modes. Quality control (QC) samples for untargeted lipidomics were prepared by centrifuging pooled lipid extracts from equal aliquots of each sample. The analytical sequence included three experimental groups, with all extraction, identification, and quantification procedures performed by Guangzhou Gidio Biotechnology Co., Ltd.

Data processing and lipid identification were performed using LipidSearch software (Version 4.1) by matching MS2 spectra against databases of phospholipids, glycerolipids, sphingolipids, and steroids, with mass accuracies of 5 ppm for precursor ions and 5 mDa for product ions. Lipid quantification based on normalized peak area (AUC) was conducted using TraceFinder software (version 4.1). After removing duplicate lipid entries, the data were log2-transformed and subjected to orthogonal partial least squares-discriminant analysis (OPLS-DA) using SIMCA software (version 14.1; Umetrics, Umea, Sweden). Differentially accumulated lipids (DALs) were screened based on variable importance in projection (VIP) >1 and independent-sample *t*-test *p* < 0.05.

### 2.8 Transcriptomic and lipid metabolomic integration analysis

The Spearman correlation coefficient (SPCC) was calculated to analyze the association between candidate gene FPKM expression levels and metabolite contents, including key fatty acids and DALs. Gene-metabolite pairs with |SPCC| >0.5 and *p* < 0.05 were selected for further investigation.

### 2.9 Statistical analysis

The results are presented as means ± standard errors, with statistical significance defined as *p* < 0.05. The experimental data were statistically analyzed using SPSS 26.0 software (IBM Corp., Armonk, NY, United States) with one-way ANOVA.

## 3 Results

### 3.1 Effects of res and HMB supplementation on intermuscular fatty acids in Tibetan sheep

Gas chromatography (GC-6890) analysis revealed significant differences (*p* < 0.05, Table 3) in the fatty acid composition of intramuscular fat among treatment groups (H, H-RES, H-HMB, H-RES-HMB). Among SFAs, C17:0 and C18:0 in the H and H-HMB groups were significantly higher than in the H-RES-HMB group (*p* < 0.05).

**Table 3:** Fatty acid profile (μg/g) in subcutaneous fat.

Items/(μg/g)	Groups				*p*-value
	H	H-RES	H-HMB	H-RES-HMB	
SFA					
C8:0	33.41 ± 0.96	41.50 ± 4.23	37.87 ± 3.76	37.18 ± 2.55	0.399
C10:0	716.42 ± 157.20	1013.35 ± 95.05	714.65 ± 36.88	647.34 ± 111.47	0.160
C11:0	53.12 ± 3.98	76.15 ± 10.48	52.64 ± 1.88	53.05 ± 2.66	0.053
C12:0	5034.02 ± 544.77	6189.08 ± 1178.96	3211.06 ± 442.58	4180.19 ± 549.86	0.099
C13:0	202.55 ± 25.85	245.23 ± 33.38	175.12 ± 13.36	164.30 ± 3.64	0.121
C14:0	54351.85 ± 4516.49	60014.67 ± 5975.76	45225.46 ± 4931.24	40688.19 ± 2840.15	0.072
C15:0	4980.16 ± 535.04	5234.81 ± 659.15	4411.98 ± 521.52	3443.59 ± 174.80	0.137
C16:0	149213.66 ± 7553.15	140675.47 ± 6162.52	131516.30 ± 10479.03	129010.03 ± 5711.23	0.303
C17:0	9095.03 ± 998.25^a^	8153.12 ± 1543.44^ab^	10614.11 ± 421.67^a^	5605.90 ± 434.10^b^	0.035
C18:0	171223.11 ± 3888.40^a^	129451.31 ± 35778.87^ab^	157723.63 ± 17932.48^a^	80958.85 ± 9003.62^b^	0.049
C20:0	2174.66 ± 331.48	1937.53 ± 145.85	2120.18 ± 393.89	1323.13 ± 127.25	0.190
C21:0	184.38 ± 31.92	181.24 ± 12.46	192.32 ± 32.14	129.56 ± 5.61	0.296
C22:0	417.36 ± 81.84	443.48 ± 36.31	409.51 ± 65.91	306.84 ± 17.71	0.390
C23:0	155.57 ± 21.89	146.58 ± 11.38	159.56 ± 19.30	120.43 ± 1.18	0.351
C24:0	238.33 ± 21.59	224.59 ± 13.45	237.15 ± 15.89	203.09 ± 2.10	0.375
MUFA					
C14:1N5	1917.28 ± 539.66	4021.86 ± 1591.94	1770.40 ± 776.67	1578.67 ± 208.36	0.287
C15:1N5	803.61 ± 94.09^b^	1055.29 ± 77.31^b^	979.92 ± 114.85^b^	1381.97 ± 72.86^a^	0.012
C16:1N7	15783.27 ± 3646.81	27792.70 ± 8128.46	19222.14 ± 6114.82	16833.66 ± 746.11	0.436
C17:1N7	4054.03 ± 817.29	5430.84 ± 1101.13	4883.13 ± 1285.73	4244.81 ± 316.94	0.734
C18:1N9	184666.95 ± 18219.74^b^	275760.53 ± 12857.53^a^	261694.16 ± 16952.94^a^	265728.49 ± 3246.92^a^	0.006
C20:1N9	1797.02 ± 224.65	2585.53 ± 208.85	2184.98 ± 321.33	2438.26 ± 157.76	0.173
C22:1N9	1090.54 ± 285.70	1538.91 ± 70.55	1323.07 ± 304.47	1280.17 ± 192.37	0.618
C24:1N9	224.51 ± 7.28	244.64 ± 17.04	246.03 ± 8.94	243.29 ± 21.40	0.714
PUFA					
C18:2N6	9007.42 ± 574.89^c^	15170.74 ± 821.29^a^	12395.49 ± 552.47^b^	16320.95 ± 233.71^a^	0.000
C18:3N6	219.10 ± 8.82^b^	298.33 ± 14.29^a^	247.87 ± 11.16^b^	297.90 ± 19.27^a^	0.009
C18:3N3	1651.85 ± 195.22^b^	2469.61 ± 73.86^a^	2198.40 ± 156.19^ab^	2712.00 ± 214.01^a^	0.011
C20:2N6	396.77 ± 29.48	521.84 ± 91.33	457.20 ± 72.75	515.97 ± 29.89	0.486
C20:3N6	191.23 ± 15.21^b^	242.65 ± 22.37^ab^	212.18 ± 14.00^b^	283.24 ± 18.77^a^	0.031
C20:4N6	412.72 ± 66.88	400.99 ± 92.06	418.58 ± 74.19	598.63 ± 93.64	0.338
C20:3N3	113.85 ± 0.77^c^	127.86 ± 5.64^ab^	121.36 ± 0.94^bc^	132.62 ± 3.32^a^	0.019
C20:5N3	128.62 ± 11.00	110.66 ± 9.96	121.18 ± 7.98	154.09 ± 19.41	0.183
C22:2N6	113.19 ± 2.11	121.55 ± 6.12	119.67 ± 5.86	121.87 ± 4.12	0.578
C22:4N6	156.75 ± 8.36	159.71 ± 15.48	173.55 ± 15.54	184.01 ± 11.00	0.459
C22:5N6	74.15 ± 2.60	75.03 ± 4.09	78.35 ± 5.09	79.72 ± 0.50	0.653
C22:6N3	142.48 ± 7.37	128.69 ± 6.94	137.89 ± 2.92	143.85 ± 1.95	0.256

Bitterness (C8:0), capric acid (C10:0), undecanoic acid (C11:0), lauric acid (C12:0), tridecanoic acid (C13:0), myristic acid (C14:0), pentadecanoic acid (C15:0), palmitic acid (C16:0), heptadecanoic acid (C17:0), stearic acid (C18:0), arachicotic acid (C20:0), docosanoic acid (C21:0), behenic acid (C22:0), docosanoic acid (C23:0), docosanoic acid (C24:0), myrcene (C14:1N5), pentacenoic acid (C15:1N5), palmitoleic acid (C16:1N7), heptacenoic acid (C17:1N7), oleic acid (C18:1N9), eicosacenoic acid (C20:1N9), erucic acid (C22:1N9), docoselenic acid (C24:1N9), linoleic acid (C18:2N6), γ-linolenic acid (C18:3N6), α-linolenic acid (C18:3N3), eicosadienoic acid (C20:2N6), eicosatrienoic acid (C20:3N6), arachidonic acid (C20:4N6), eicosatrienoic acid (C20:3N3), eicosapentaenoic acid (C20:5N3), docosadienoic acid (C22:2N6), docosatetraenoic acid (C22:4N6), docosapentaenoic acid (C22:5N6), docosahexaenoic acid (C22:6N3). ^*^p < 0.05 indicates significant difference.

For MUFAs, C15:1n5 in the H-RES-HMB group was significantly higher than in the H, H-RES, and H-HMB groups (*p* < 0.05), while C18:1n9 in the H-RES, H-HMB, and H-RES-HMB groups was significantly higher than in the H group (*p* < 0.05). Among PUFAs, C18:2n6 in the H-RES and H-RES-HMB groups was significantly higher than in the H and H-HMB groups (*p* < 0.05), with the H-HMB group also exhibiting higher C18:2n6 than the H group (*p* < 0.05). C18:3n6 in the H-RES and H-RES-HMB groups was significantly higher than in the H and H-HMB groups (*p* < 0.05). C18:3n3 in the H-RES and H-RES-HMB groups was significantly higher than in the H group (*p* < 0.05). C20:3n6 and C20:3n3 in the H-RES-HMB group were significantly higher than in the H and H-HMB groups (*p* < 0.05), while C20:3n3 in the H-RES group was also significantly higher than in the H group (*p* < 0.05).

### 3.2 Effects of supplementation with RES and HMB on the flavor characteristics of Tibetan sheep meat

In this study, gas chromatography-ion mobility spectrometry (GC-IMS) was successfully employed for the accurate identification of volatile flavor compounds in Tibetan sheep meat. As shown in Table 4, the contents of 2-hexanone, 3-hexanone, and methyl acetate in all experimental groups (H-RES and H-HMB groups) were significantly higher than those in the control group (H group) (*p* < 0.05), with the H-RES-HMB group exhibiting significantly higher levels than the other two groups (*p* < 0.05). For 3-pentanone, the H-HMB and H-RES-HMB groups showed significantly higher contents than the H and H-RES groups (*p* < 0.05). The control group (H group) had significantly higher levels of 3-methyl butanal, heptanal, and n-octanal compared to the experimental groups (H-RES, H-HMB, and H-RES-HMB groups) (*p* < 0.05).

**Table 4:** Flavor characteristic indices of Tibetan sheep meat.

Items	Groups				*p*-value
	H	H-RES	H-HMB	H-RES-HMB	
2-Heptanone	50.55 ± 7.22	52.99 ± 1.70	59.67 ± 3.54	40.56 ± 2.08	0.070
2-Hexanone	45.18 ± 4.97^d^	83.97 ± 0.98^b^	64.01 ± 1.18^c^	128.86 ± 2.83^a^	0.001
3-Hexanone	47.77 ± 3.21^d^	104.32 ± 2.84^b^	82.93 ± 1.15^c^	186.99 ± 3.34^a^	0.001
3-Pentanone	340.92 ± 4.82^b^	357.14 ± 11.98^b^	449.27 ± 26.16^a^	427.78 ± 18.33^a^	0.005
3-Methyl butanal	59.74 ± 3.95^a^	33.33 ± 0.57^b^	31.70 ± 2.49b	37.02 ± 1.38^b^	0.001
Heptanal	273.80 ± 21.45^a^	61.00 ± 2.79^b^	64.56 ± 4.28b	64.72 ± 5.22^b^	0.001
n-Octanal	83.00 ± 9.43^a^	59.14 ± 0.66^b^	61.71 ± 1.83^b^	55.06 ± 0.20^b^	0.014
2-methylpropyl butanoate	57.82 ± 8.67	56.74 ± 1.05	56.54 ± 1.78	53.29 ± 0.31	0.899
Ethyl butanoate	43.77 ± 4.23	50.73 ± 0.71	57.81 ± 5.26	57.40 ± 0.99	0.061
Methyl acetate	182.97 ± 7.18^c^	213.61 ± 1.54^b^	233.04 ± 12.26^b^	282.91 ± 5.33^a^	0.001

These 10 flavor compounds can be categorized into four groups: 2-heptanone, 2-hexanone, and 3-hexanone belong to methyl ketones; 3-methyl butanal, heptanal, and n-octanal are short-chain aldehydes; 2-methylpropyl butanoate, ethyl butanoate, and methyl acetate are esters; and 3-pentanone is classified as a special ketone. *p* < 0.05.

### 3.3 Effects of supplementation with RES and HMB on the histology of intermuscular fat in Tibetan sheep

**Figure 1:**
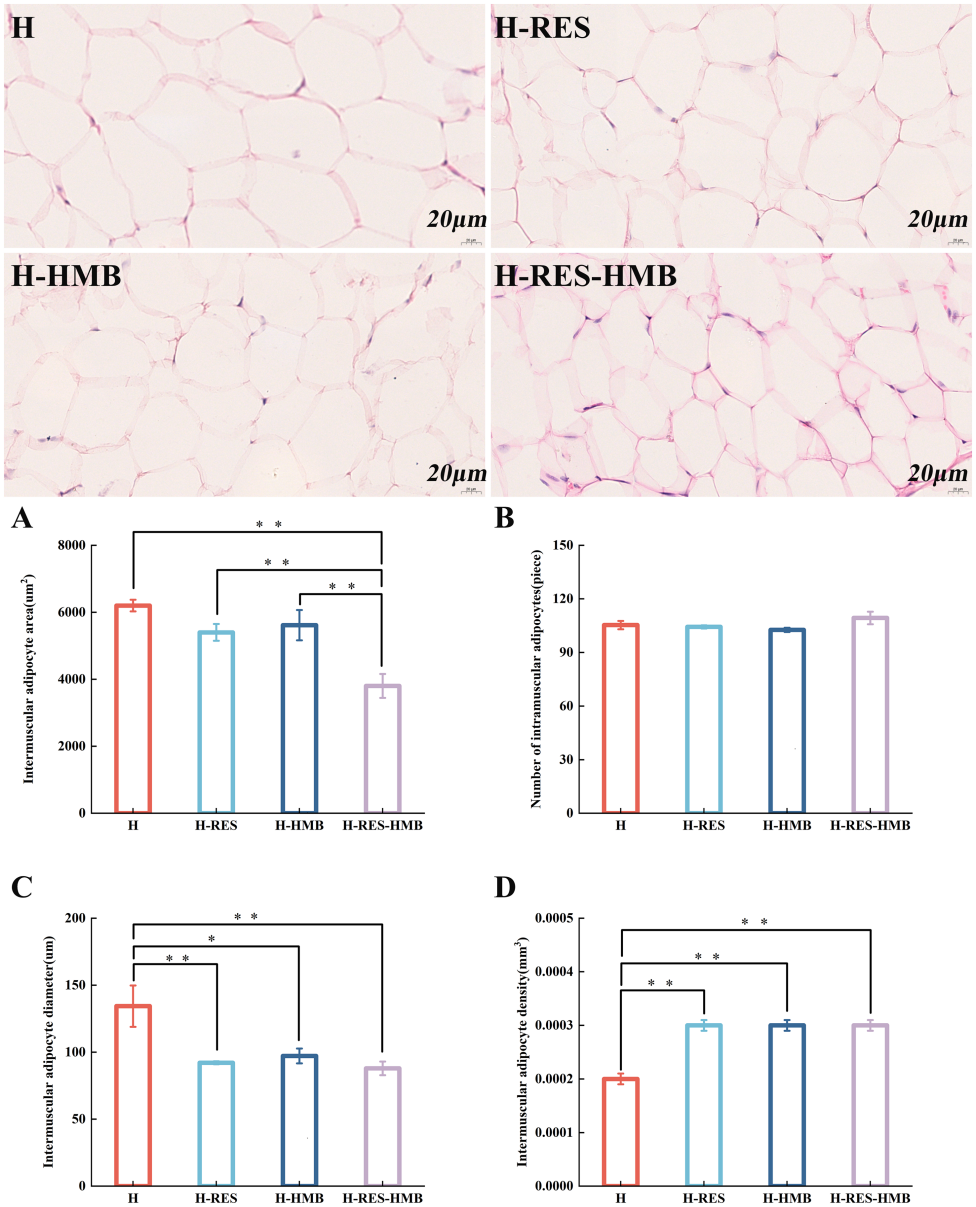
Cryosections of intramuscular adipose tissue. The H group received basal diet, H-RES group received basal diet + resveratrol (1.5 g/day), H-HMB group received basal diet + β-hydroxy-β-methylbutyrate (1,250 mg/day), and H-RES-HMB group received basal diet + resveratrol (1.5 g/day) + β-hydroxy-β-methylbutyrate (1,250 mg/day). HE staining, 40× magnification. **(A)** Intramuscular adipocyte area. **(B)** Number of intramuscular adipocytes. **(C)** Intramuscular adipocyte diameter. **(D)** Intramuscular adipocyte density. “**” indicates extremely significant difference (*p* < 0.01), “*” indicates significant difference (*p* < 0.05), and absence of “*” indicates no significant difference (*p* > 0.05).

Histological examination of HE-stained sections under 40× light microscopy revealed that the intramuscular adipocyte areas in the H, H-RES, and H-HMB groups were significantly larger than those in the H-RES-HMB group (*p* < 0.01) (Figure 1A). The adipocyte diameter in the H group was markedly greater than that in both the H-RES and H-RES-HMB groups (*p* < 0.01), while being significantly larger than the H-HMB group (*p* < 0.05) (Figure 1C). The adipocyte densities in the H-RES, H-HMB, and H-RES-HMB groups were all significantly higher than that in the H group (*p* < 0.01) (Figure 1D). Microscopic examination revealed that all four experimental groups (Figures 1A–D) maintained normal adipose tissue morphology. Remarkably, the H-RES-HMB group demonstrated the smallest adipocyte area and diameter, along with the highest cell density values, with all these differences showing statistical significance.

### 3.4 Identification and functional enrichment analysis of differentially expressed genes

**Figure 2:**
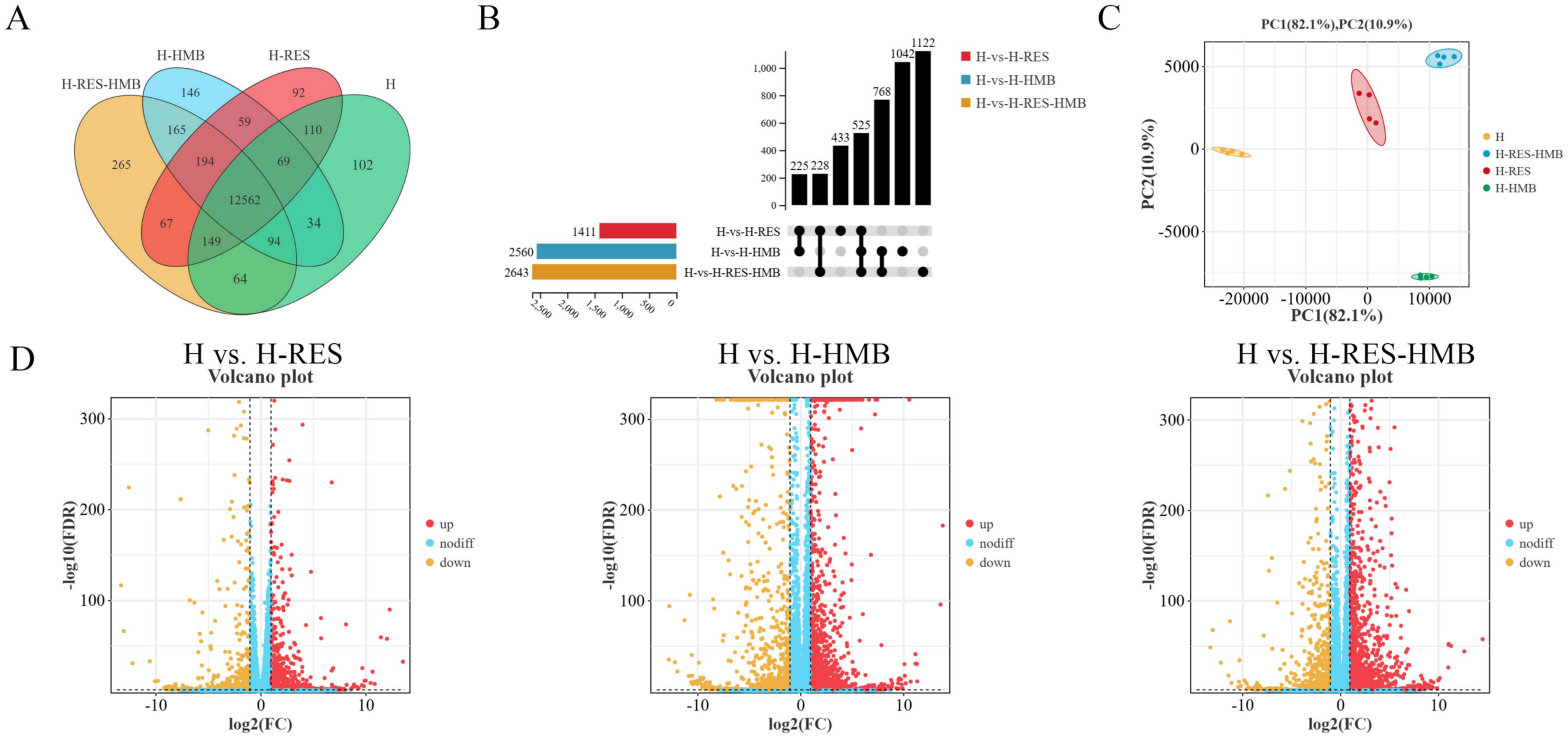
**(A)** Venn diagram showing shared (overlapping) and unique (non-overlapping) genes between groups. **(B)** UpSet plot visualizing multi-group intersections, overcoming Venn diagram limitations for >5 groups. **(C)** PCA plot of gene expression data evaluating intra-group consistency and inter-group differences. **(D)** Volcano plot displaying DEGs in pairwise comparisons, with extreme x-axis positions indicating larger fold changes (red = upregulated, green = downregulated, blue = non-significant).

To investigate the regulatory mechanisms of RES and HMB on intermuscular fat deposition in Tibetan sheep, this study performed transcriptome analysis of intermuscular adipose tissue from experimental and control groups using RNA-seq technology. After quality control filtering, the H, H-RES, H-HMB, and H-RES-HMB groups obtained 17.12, 15.79, 15.19, and 15.22 million high-quality clean reads, respectively, with mapping rates >91% for all samples (reference genome: sheep Oar_v4.03, Supplementary Table 1). Differential gene analysis results in Figure 2A showed that the H, H-RES, H-HMB, and H-RES-HMB groups contained 102, 92, 146, and 265 specifically expressed genes, respectively, while 12,562 genes were co-expressed across all four groups. The UpSet plot (Figure 2B) indicated that the H vs. H-RES-HMB group had the highest number of differentially expressed genes (2,643), with 525 shared differentially expressed genes among the three comparison groups. PCA analysis revealed that PC1 and PC2 explained 82.1 and 10.9% of the variance, respectively, with the two principal components covering 93% of the variance. The scatter points of the H-RES-HMB group were relatively more concentrated within the same quadrant (Figure 2C). Volcano plots further demonstrated that the H vs. H-RES, H vs. H-HMB, and H vs. H-RES-HMB comparisons detected 1,411 (828 upregulated, 583 downregulated), 2,560 (1,589 upregulated, 971 downregulated), and 2,643 (1,844 upregulated, 799 downregulated) differentially expressed genes, respectively (Figure 2D).

**Figure 3:**
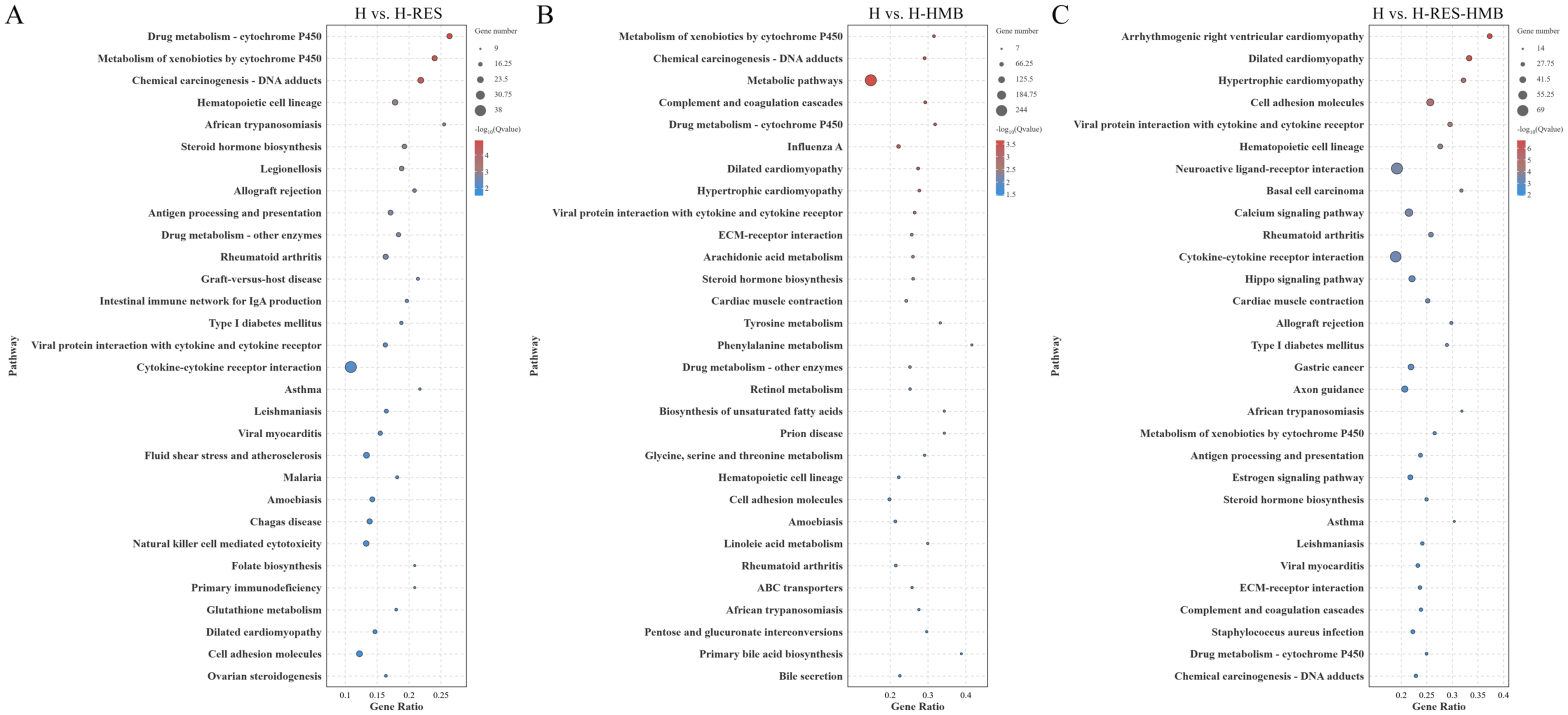
KEGG pathway enrichment analysis was conducted on the intermuscular adipose tissues of Tibetan sheep across three comparison groups: **(A)** H vs. H-RES, **(B)** H vs. H-HMB, and **(C)** H vs. H-RES-HMB. Bubble plots illustrate the top 30 significantly enriched pathways, with the y-axis indicating the KEGG pathway names.

To further elucidate the functional characteristics of differentially expressed genes (DEGs), we performed systematic functional enrichment analysis. KEGG pathway analysis (Supplementary Table 2) revealed that the H vs. H-RES comparison was significantly enriched in 320 pathways, H vs. H-HMB in 335 pathways, and H vs. H-RES-HMB in 326 pathways. In the differential KEGG enrichment analysis (Figure 3), four significant pathways (*p* < 0.05) were commonly identified in both the H vs. H-HMB and H vs. H-RES-HMB comparisons, including the Calcium signaling pathway (*ERBB4*, *P2RX7*, *ERBB3*, *P2RX3*, and *SLC8A1*), Hippo signaling pathway (*WNT9A*, *WNT10B*, *WNT6*, and *WNT2B*), Estrogen signaling pathway (*HSP90AA1*, *TGFA*, and *RARA*), and Arachidonic acid metabolism (*PLA2G4A*, *ALOX12*, and *PTGDS*). Notably, these pathways were also significantly enriched in the H vs. H-RES comparison, and all four are closely associated with the regulation of lipid metabolism.

**Figure 4:**
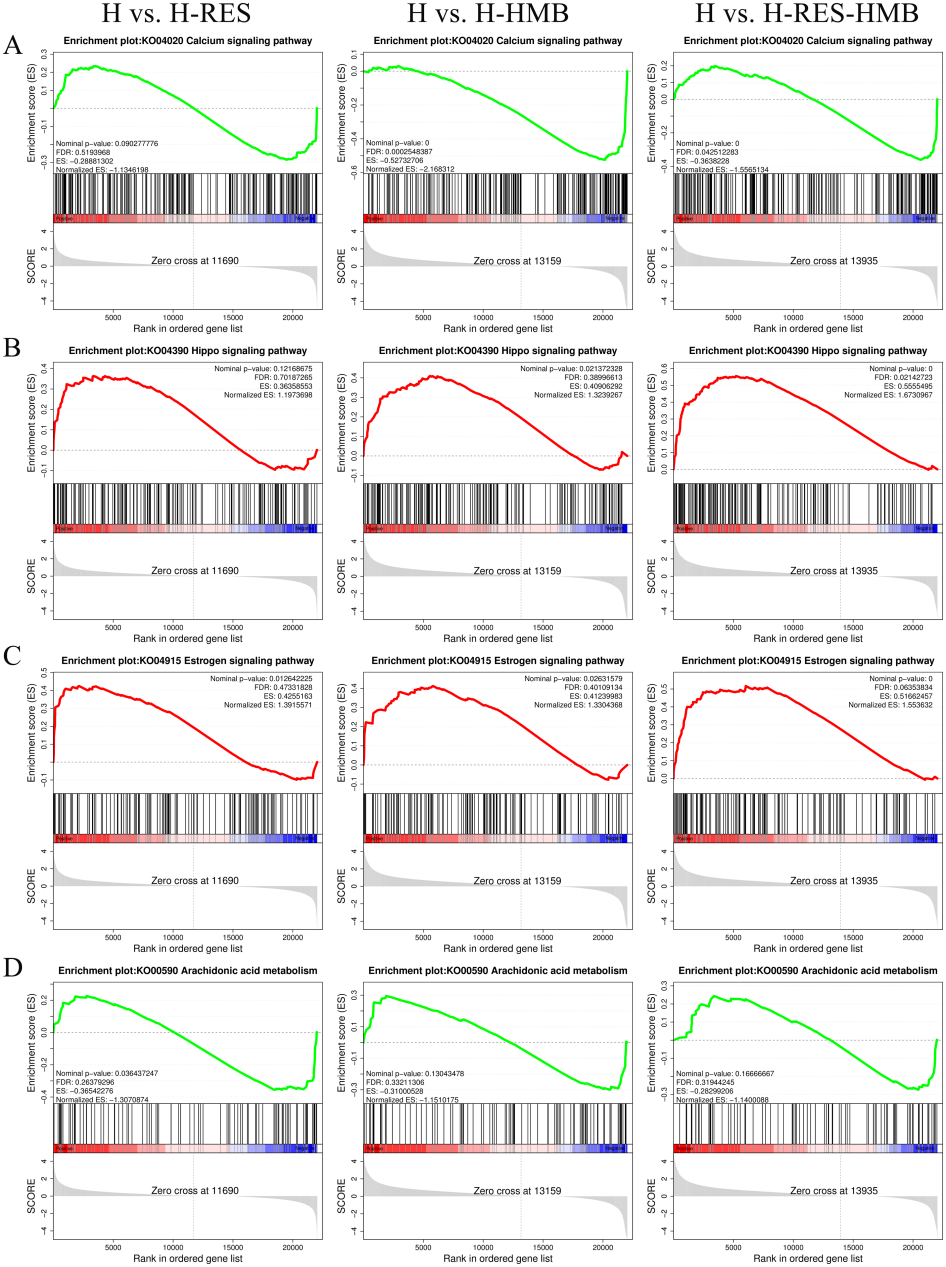
In this study, through GSEA enrichment analysis, the differentially expressed genes (DEGs) in the intermuscular adipose tissue of Tibetan sheep in the H group (control group), H-RES group, H-HMB group, and H-RES-HMB group (experimental groups) were compared, and the following key signaling pathways were identified. **(A)** Calcium signaling pathway. **(B)** Hippo signaling pathway. **(C)** Estrogen signaling pathway. **(D)** Arachidonic acid metabolism.

Gene set enrichment analysis (GSEA) results demonstrated that the Calcium signaling pathway (Figure 4A) and Arachidonic acid metabolism (Figure 4D) (with negative ES values) exhibited higher activity in the control group (H), whereas the Hippo signaling pathway (Figure 4B) and Estrogen signaling pathway (Figure 4C) (with positive ES values) were significantly activated in the treatment groups (H-RES, H-HMB, and H-RES-HMB). Differential expression analysis further revealed that 15 lipid metabolism-related genes, including *ERBB4* and *P2RX7*, were significantly upregulated (*p* < 0.05) in the treatment groups (H-RES, H-HMB, and H-RES-HMB) compared to the control group (H).

**Figure 5:**
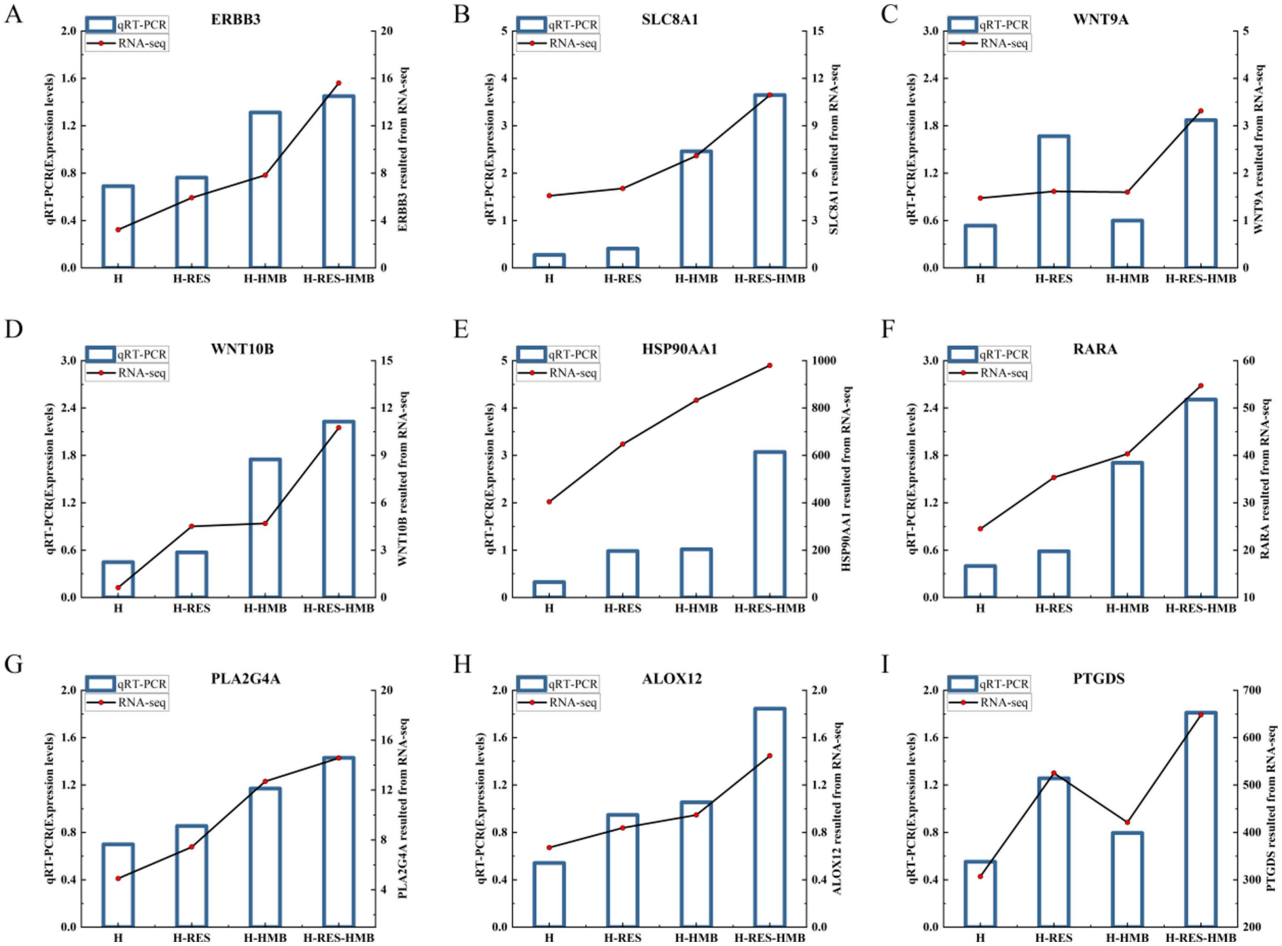
Confirmation of the expression patterns of the nine selected genes via the qRT-PCR. The qRT-PCR results were consistent with the RNA-seq data. **(A)** *ERBB3*. **(B)** *SLC8A1*. **(C)** *WNT9A*. **(D)** *WNT10B*. **(E)** *HSP90AA1*. **(F)** *RARA*. **(G)** *PLA2G4A*. **(H)** *ALOX12*. **(I)** *PTGDS*.

To validate the reliability of the RNA-seq results, we selected nine genes for expression level verification using quantitative reverse transcription polymerase chain reaction (qRT-PCR). The mRNA expression levels of all genes were consistent with the RNA-seq data, confirming the reliability of the RNA-seq results (Figure 5).

### 3.5 Data analysis of the liposomes in the intermuscular fat

**Figure 6:**
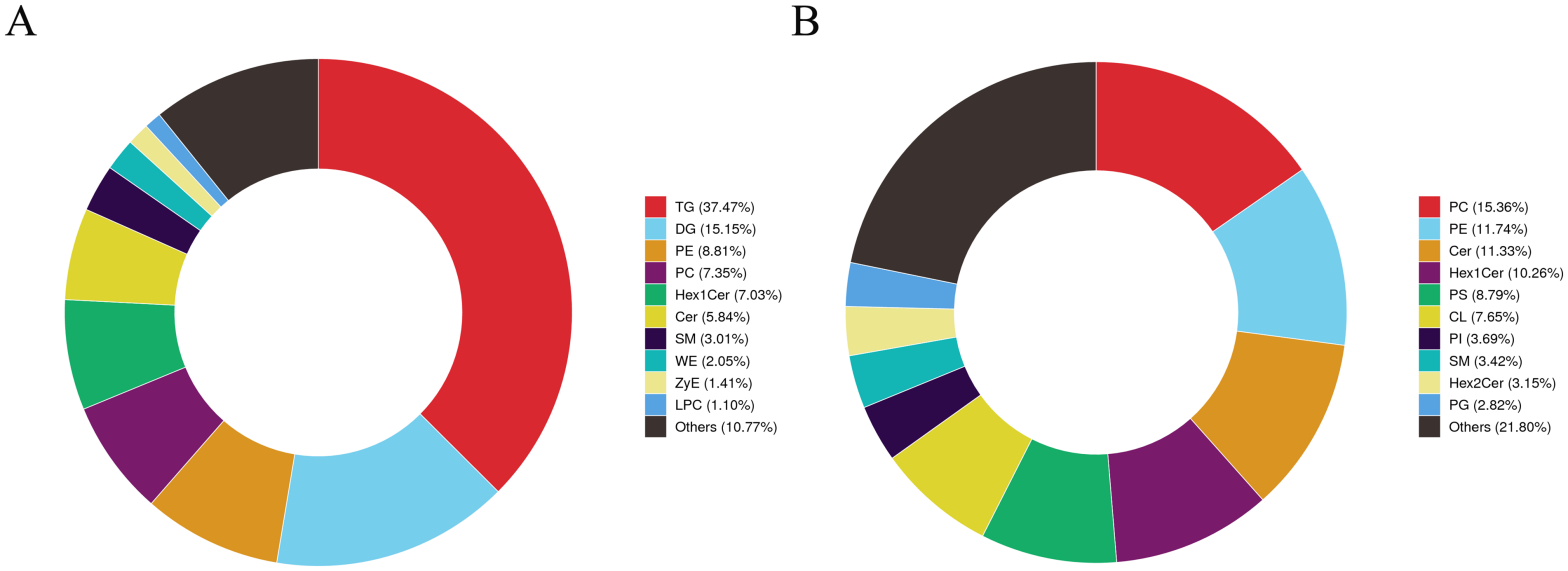
Qualitative and quantitative analysis of intermuscular lipids in Tibetan sheep was conducted in the H vs. H-RES, H vs. H-HMB, and H vs. H-RES-HMB groups. **(A)** Statistical chart of POS ion lipid composition. **(B)** Statistical chart of NEG ion lipid composition.

Based on the previous findings that intermuscular fat deposition significantly affects the meat quality of Tibetan sheep, this study systematically identified lipid species and content through lipidomics: first, lipid molecules were characterized using UPLC-HRMS, followed by multivariate statistical analysis to reveal differences in lipid molecules among different treatment groups. This study examined the lipid metabolic characteristics of intermuscular adipocytes and explored the metabolic-related networks of the H vs. H-RES, H vs. H-HMB, and H vs. H-RES-HMB groups. Based on high-throughput LC-MS analysis with an adjusted *p*-value threshold of less than 0.05, a total of 499 POS-positive ion lipid metabolites and 690 NEG-positive ion lipid metabolites were detected (Supplementary Table 3). As shown in Figure 6A, in the POS ion lipid composition statistics, except for the “Others” category (10.77%), TG accounted for the largest proportion (37.47%), while LPC accounted for the smallest proportion (1.10%). Similarly, Figure 6B shows that in the NEG ion lipid composition statistics, except for the “Others” category (21.80%), PC accounted for the largest proportion (15.36%), while PG accounted for the smallest proportion (2.82%).

**Figure 7:**
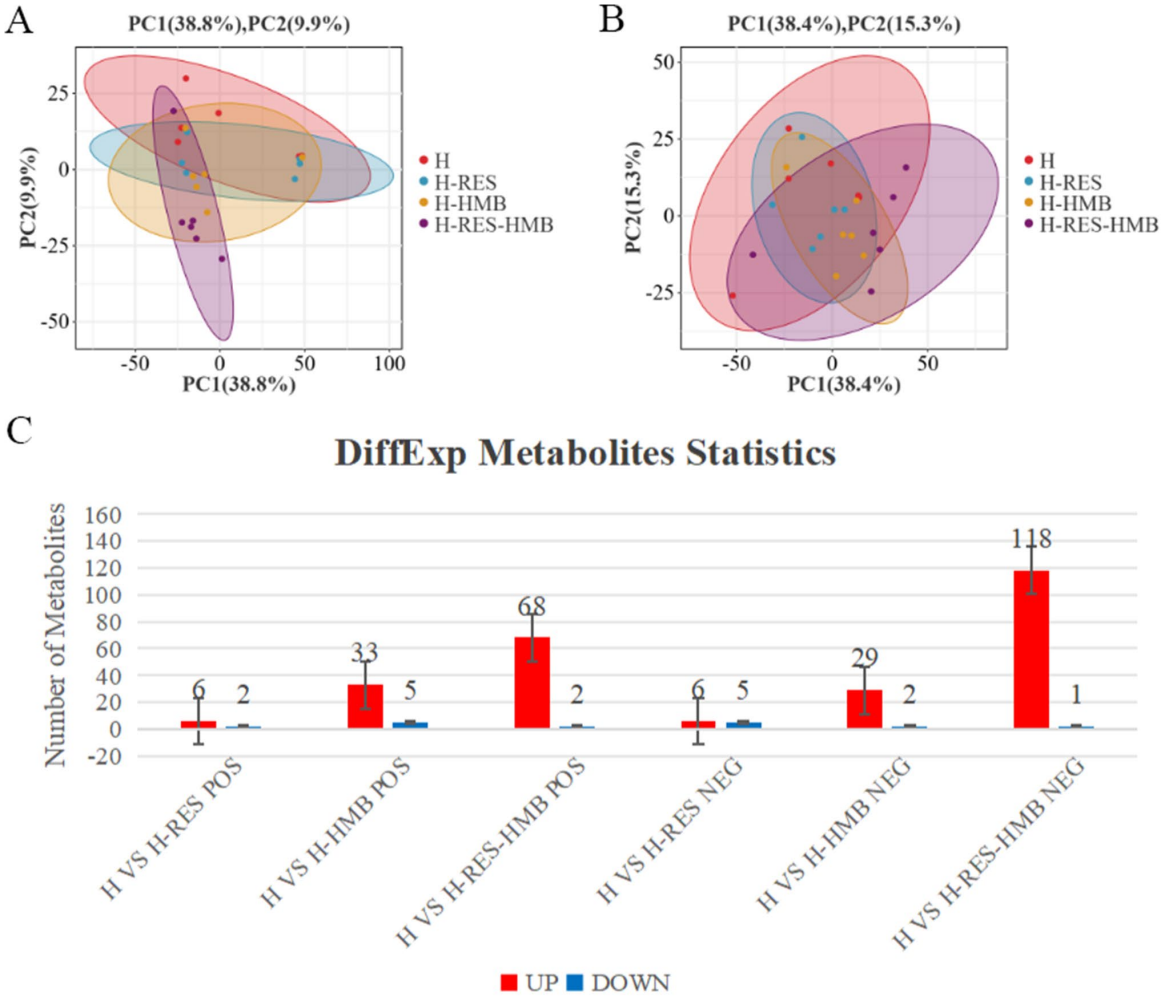
**(A)** Multivariate statistical PCA plot of positive ion mode for H, H-RES, H-HMB, and H-RES-HMB groups. **(B)** Multivariate statistical PCA plot of negative ion mode for H, H-RES, H-HMB and H-RES-HMB groups. **(C)** Number of up-regulated and down-regulated metabolites in different comparison groups under POS and NEG modes.

**Figure 8:**
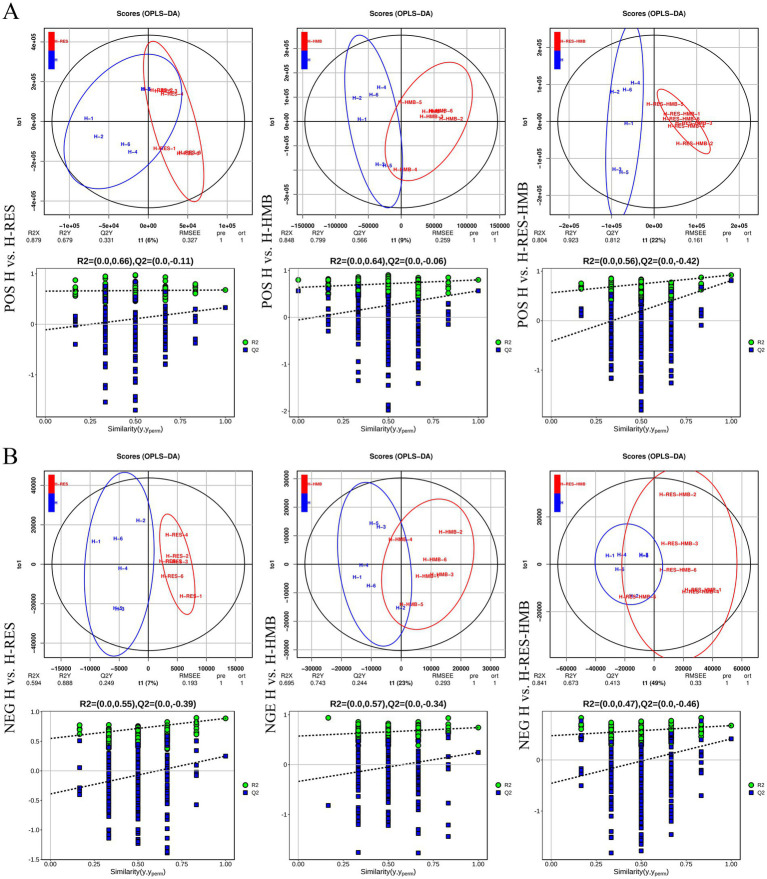
Orthogonal partial least squares-discriminant analysis (OPLS-DA) scores of lipid metabolites in intramuscular fat. **(A)** The OPLS-DA models and score plots for all three sample groups were positive. **(B)** The OPLS-DA models and score plots for all three sample groups showed negative results.

**Figure 9:**
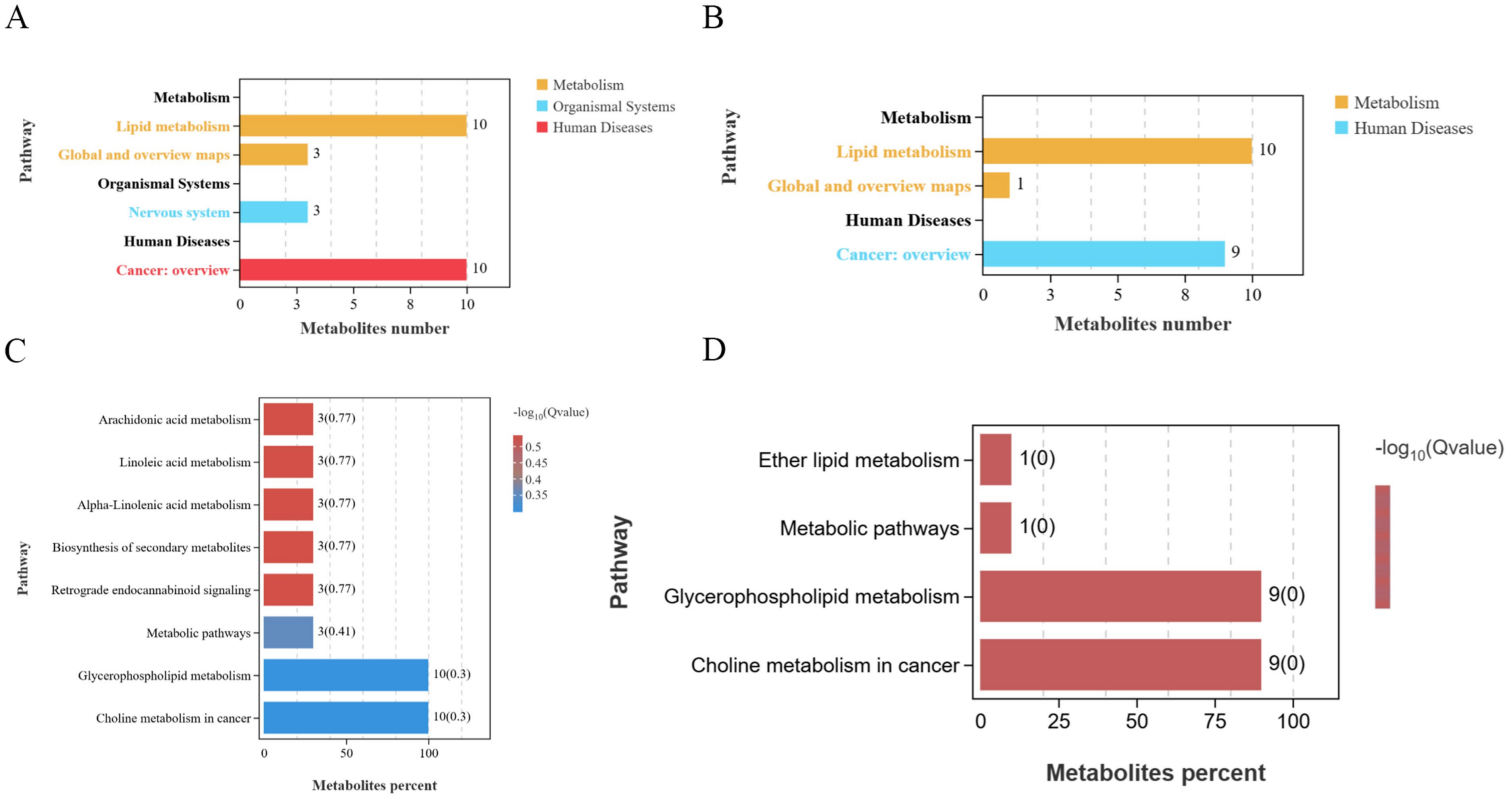
**(A)** KEGG positive ion count statistics chart. **(B)** KEGG negative ion count statistics chart. **(C)** KEGG positive ion enrichment bar plot. **(D)** KEGG negative ion enrichment bar plot. Differential metabolites were annotated in the KEGG database, followed by pathway enrichment analysis, with significantly enriched metabolic pathways (*p* < 0.05) identified based on hypergeometric tests.

PCA results revealed clear separation among the three groups, including quality control samples, in the principal component (PC1 × PC2) score plots (Figures 7A,B). A total of 277 differential metabolites were identified (260 in positive mode and 17 in negative mode, Figure 7C). Multivariate analysis of subcutaneous fat metabolites showed that in the H-RES group, 19 differential metabolites exhibited significant changes (12 upregulated and 7 downregulated). In the H-HMB group, 69 differential metabolites were significantly altered (62 upregulated and 7 downregulated), while in the H-RES-HMB group, 189 differential metabolites showed significant changes (186 upregulated and 3 downregulated). OPLS-DA plots and permutation test plots confirmed substantial differences in metabolites among the three groups of Tibetan sheep intermuscular fat, demonstrating the accuracy and reproducibility of the LC-MS/MS method (Figures 8A,B). Generally, *R*^2^ and *Q*^2^ should exceed 0.5, with a difference of less than 0.3 between them. Under type/POS and type/NEG ion modes, t1 accounted for the largest proportions (22 and 49%, respectively). In the type/POS mode, the highest number of significantly altered metabolites in the H vs. H-RES group were TGs (45 species, 42.0%), while in the H vs. H-HMB group, TGs were also predominant (79 species, 38.5%). Similarly, in the H vs. H-RES-HMB group, TGs showed the most significant changes (135 species, 34.4%). Under the Type/NEG mode, CL and PC were the most altered metabolites in the H vs. H-RES group (11 species each, 20.0%), whereas PC and PS were predominant in the H vs. H-HMB group (24 species each, 14.1%). In contrast, Hex1Cer exhibited the highest number of changes in the H vs. H-RES-HMB group (102 species, 21.9%). Thus, it was concluded that TGs were the most abundant in the Type/POS mode, whereas Hex1Cer dominated in the type/NEG mode (lipid KEGG statistics, Supplementary Table 4). In Figure 9 and Supplementary Table 5, lipid metabolism was enriched in pathways such as glycerophospholipid metabolism, Arachidonic acid metabolism, linoleic acid metabolism, alpha-linolenic acid metabolism, and ether lipid metabolism. Global and overview maps were enriched in metabolic pathways and biosynthesis of secondary metabolites, while the nervous system was associated with retrograde endocannabinoid signaling. Cancer: overview was linked to choline metabolism in cancer. Metabolites in these pathways were predominantly LPCs and PCs, with five key metabolites identified in lipid metabolism: LPC (20:0), LPC (20:1), PC (21:2), PC (37:0), and PC (38:5).

### 3.6 Integrated transcriptomic and lipid metabolomic analysis

**Figure 10:**
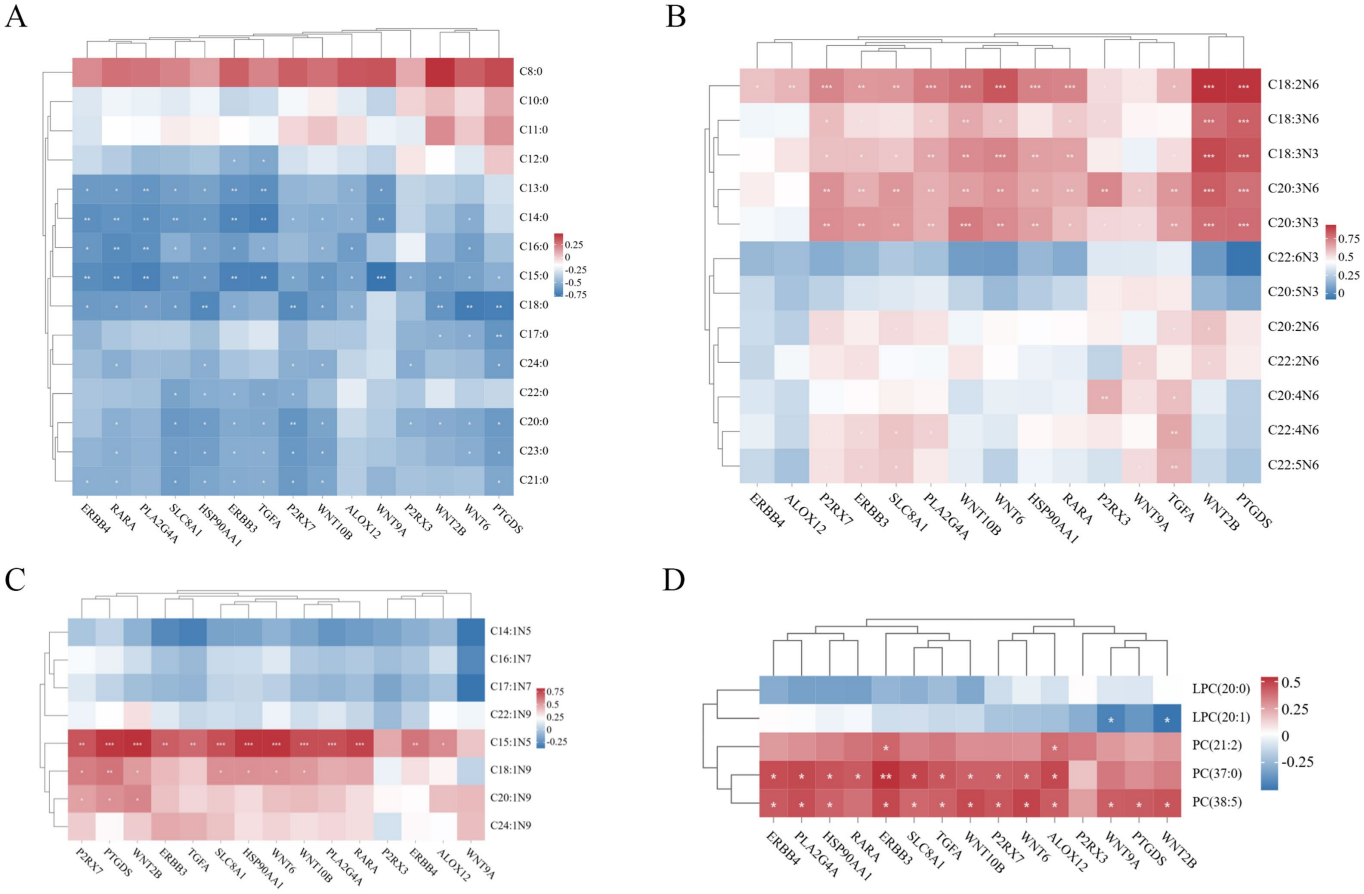
**(A)** Heatmap of gene-saturated fatty acid (SFA) association analysis. **(B)** Heatmap of gene-polyunsaturated fatty acid (PUFA) association analysis. **(C)** Heatmap of gene-monounsaturated fatty acid (MUFA) association analysis. **(D)** Heatmap of gene-lipid metabolism compound association analysis. The color scale indicates correlation coefficients, with red and blue representing positive and negative correlations, respectively. Asterisks denote significance levels: ^*^p < 0.05, ^**^p < 0.01, and ^***^p ≤ 0.001.

Spearman correlation analysis was performed to calculate correlation coefficients between the expression levels of 15 candidate genes and medium- and long-chain fatty acid content as well as key lipid metabolites, enabling integrated analysis of transcriptomic and lipid metabolomic data. In Figure 10A, multiple genes exhibited significant negative correlations with saturated fatty acids (SFAs). ERBB4, RARA, *PLA2G4A*, *SLC8A1*, *HSP90AA1*, *ERBB3*, *TGFA*, *P2RX7*, *WNT10B*, *ALOX12*, *WNT9A*, *P2RX3*, *WNT2B*, *WNT6*, and *PTGDS* showed inverse associations with specific SFA chain lengths (C12:0–C24:0). Notably, *HSP90AA1* correlated with the broadest range (C13:0–C24:0), while *WNT9A* and *P2RX3* were linked to fewer SFAs (e.g., C13:0–C15:0 and C15:0/C20:0/C24:0, respectively). In Figure 10B, Several genes displayed significant positive correlations with polyunsaturated fatty acids (PUFAs). ERBB4 was linked to C18:2N6, while *ALOX12* correlated with C18:2N6 and C18:3N3. *P2RX7*, *ERBB3*, *SLC8A1*, and *PLA2G4A* showed broad associations, spanning C18:2N6 to C22:5N6. *WNT10B*, *WNT6*, *HSP90AA1*, *RARA*, and *PTGDS* shared correlations with C18:2N6–C20:3N3, whereas *P2RX3* and *WNT9A* were linked to longer-chain PUFAs (e.g., C20:4N6, C22:5N6). *TGFA* and *WNT2B* also exhibited strong PUFA associations, particularly with C18:2N6–C22:2N6.

In Figure 10C, genes (*P2RX7*, *PTGDS*, *WNT2B*, *ERBB3*, *TGFA*, *SLC8A1*, *HSP90AA1*, *WNT6*, *WNT10B*, *PLA2G4A*, *RARA*, *ERBB4*, and *ALOX12*) showed significant positive correlations with MUFAs (C15:1N5), while genes (*P2RX7*, *PTGDS*, *WNT2B*, *SLC8A1*, *HSP90AA1*, *WNT6*, and *WNT10B*) were positively correlated with MUFAs (C18:1N9), and genes (*P2RX7*, *PTGDS*, and *WNT2B*) exhibited positive correlations with MUFAs (C20:1N9). In Figure 10D, genes (*WNT9A* and *WNT2B*) demonstrated significant negative correlations with LPC (20:1), whereas genes (*ERBB3* and *ALOX12*) showed positive correlations with the lipid metabolite PC (21:2). Furthermore, genes (*ERBB4*, *PLA2G4A*, *HSP90AA1*, *RARA*, *ERBB3*, *SLC8A1*, *TGFA*, *WNT10B*, *P2RX7*, *WNT6*, and *ALOX12*) were positively correlated with PC (37:0), and genes (*ERBB4*, *PLA2G4A*, *HSP90AA1*, *ERBB3*, *SLC8A1*, *TGFA*, *WNT10B*, *P2RX7*, *WNT6*, *ALOX12*, *WNT9A*, *PTGDS*, and *WNT2B*) showed positive associations with PC (38:5).

## 4 Discussion

This study revealed that the combined RES and HMB treatment significantly regulated intramuscular fat (IMF) deposition in Tibetan sheep by modulating key morphological characteristics including IMF area, density, and adipocyte diameter, providing a novel strategy for improving ruminant meat quality. As an essential adipose tissue distributed between muscle fiber bundles, appropriate IMF deposition plays a critical role in meat quality by promoting flavor compound accumulation and significantly enhancing tenderness (24). Our results demonstrated that the combined treatment group (H-RES-HMB) significantly reduced adipocyte area and diameter while increasing adipocyte density, with all treatment groups maintaining normal physiological ranges of adipose tissue morphology. At the molecular level, RES primarily inhibits adipocyte differentiation by activating the AMPK/SIRT1 signaling pathway to suppress key adipogenic transcription factors PPARγ (25), whereas HMB promotes muscle protein synthesis through the mTOR pathway, indirectly altering the muscle fiber microenvironment and limiting the proliferative space of adipocyte precursors (26). These compounds exhibit synergistic effects by simultaneously upregulating lipolytic enzymes (ATGL and HSL) while inhibiting fatty acid synthase (FAS) and acetyl-CoA carboxylase (ACC) expression (11, 27). This dual regulation not only reduces existing adipocyte size but also promotes the differentiation of adipocyte precursors into smaller adipocytes. Ultimately, this precise modulation achieves optimized IMF distribution, maintaining meat juiciness and flavor compound deposition while establishing new theoretical foundations and practical approaches for ruminant meat quality improvement (28, 29).

This study systematically elucidates the synergistic regulatory mechanisms of combined RES and HMB supplementation on meat quality. Our findings demonstrate that the combination significantly reduces long-chain saturated fatty acids (SFA, C17:0 and C18:0) content, where these SFAs (C14:0-C18:0) inhibit intramuscular adipocyte proliferation but promote hypertrophy through the TLR4/NF-κB pathway, ultimately reducing IMF deposition (30, 31). RES activates AMPK to enhance fatty acid oxidation (32), while HMB suppresses SREBP-1c to inhibit fatty acid synthesis (33), synergistically attenuating SFA production and accumulation (34, 35). Notably, the combined treatment markedly increased monounsaturated (MUFA, C15:1N5 and C18:1N9) and polyunsaturated fatty acid (PUFA, C18:2N6, C18:3N6, C18:3N3, C20:3N6 and C20:3N3) content. These UFAs promote intramuscular adipocyte differentiation and lipid accumulation by modulating PPARγ and SREBP-1 expression, improving marbling scores (36, 37), while concurrently enhancing tenderness and juiciness by altering membrane phospholipid composition to reduce muscle fiber diameter, and exerting antioxidant effects to suppress proinflammatory cytokines (TNF-α, IL-6) and protein degradation (38, 39). Importantly, the combination combined effect UFA accumulation: RES upregulated SCD1 via SIRT1/AMPK/PPARγ to stimulate oleic acid (C18:1N9) synthesis (40, 41), while HMB promoted intramuscular fat deposition by inhibiting ubiquitin-proteasome system and activating mTOR pathway (11). Moreover, RES’s antioxidant capacity protected n-3/n-6 PUFAs from oxidation (42), and HMB optimized lipid metabolism by reducing inflammatory factors (43). These UFAs (particularly C18:1N9 and C18:2N6) generate flavor precursors (aldehydes, ketones) via β-oxidation (44), while DHA/EPA modulate lipoxygenase activity to influence lipid oxidation, collectively determining sensory quality and shelf-life (45). Our results demonstrate that RES-HMB combination coordinately regulates fatty acid metabolism through multi-target mechanisms, providing a novel nutritional strategy for meat quality improvement.

Meat flavor, a core indicator of meat quality, directly influences consumer acceptability and product value (46). Recent studies on lipid metabolism modulators such as Mangiferin and herbal essential oils have highlighted their roles in improving flavor-related lipidomic profiles, underscoring the potential of natural bioactives for meat quality enhancement (47, 48). Research demonstrates that volatile flavor compounds derived from fatty acid oxidation (e.g., aldehydes, ketones) are critical determinants of meat aroma and taste profiles (49). This study found that combined RES and HMB supplementation synergistically modulates lipid oxidation and microbial metabolic pathways, significantly enhancing key flavor compounds. Specifically, 2-hexanone, 3-hexanone, 3-pentanone, and methyl ketones—characteristic products of fatty acid β-oxidation—impart fruity, cheesy, or grassy notes (50, 51), while methyl acetate, a short-chain fatty acid ester with fruity-sweet characteristics, typically forms through fatty acid-alcohol esterification (52). RES preserves flavor precursors by inhibiting excessive lipid oxidation via its antioxidant properties (53), whereas HMB, as a leucine metabolite, combined effect branched-chain amino acid degradation and microbial metabolic activity (12). Their complementary actions optimize flavor precursor availability and metabolic efficiency, thereby significantly improving flavor intensity, Concurrently, the RES-HMB combination synergistically suppresses lipid peroxidation and protein degradation, markedly reducing aldehydes like 3-methylbutanal, heptanal, and n-octanal (53, 54). Differential flavor profiling revealed distinct characteristics among treatment groups: the control group (H) exhibited baseline levels of typical aldehydes [3-methylbutanal (malty/chocolate), heptanal (fatty/grassy), n-octanal (citrusy/fatty)], RES alone maintained balanced aldehyde levels via antioxidant effects (55), HMB alone enhanced 3-methyl butanal accumulation due to promoted branched-chain metabolism, intensifying fermented notes (56, 57), whereas the RES-HMB group achieved optimal flavor regulation, maintaining equilibrium while optimizing the proportions of characteristic compounds (58, 59). Collectively, RES-HMB co-supplementation synergistically enhances flavor, tenderness, and juiciness by promoting beneficial flavor compound synthesis and optimizing aldehyde composition, thereby comprehensively improving meat quality.

Transcriptomic analysis revealed molecular mechanisms underlying key signaling pathways regulating meat quality (60). This study elucidated the coordinated regulation of intramuscular fat (IMF) deposition by Calcium, Hippo, Estrogen, and Arachidonic acid metabolic pathways. The analysis demonstrated significant functional crosstalk between Calcium and Estrogen signaling pathways. Estrogen rapidly activates Calcium signaling via receptor (ER)-mediated non-genomic effects, where the estrogen-ER complex promotes eNOS phosphorylation through PI3K/AKT, increasing NO production to modulate calcium transients via S-nitrosylation of regulatory proteins (e.g., ryanodine receptors or SERCA), thereby inhibiting calcium overload-induced apoptosis (61, 62). Concurrently, Calcium signaling activation relies on receptor-gated calcium channels (e.g., *P2RX7*/*P2RX3*) to elevate intracellular Ca^2+^, forming Ca^2+^-CALM complexes that activate phosphoinositide signaling, synergizing with ER’s non-genomic effects to regulate downstream transduction (63, 64). Additionally, ER recruits coactivators (COA) via binding to Hsp90 (*HSP90AA1*) in classical genomic pathways, modulating target genes (e.g., *TGFA*, *RARA*) to integrate cellular proliferation/differentiation with calcium homeostasis, establishing a networked crosstalk between non-genomic and genomic regulation (65, 66). This estrogen-calcium crosstalk inhibits myocyte apoptosis and optimizes lipid metabolism, combined effect IMF deposition and meat quality (67). RES-HMB co-treatment activates ER-mediated Calcium signaling and RARA expression, suppressing excessive fat accumulation while maintaining ideal distribution to improve tenderness and juiciness (68, 69). Individually, RES reduces fat deposition via antioxidant and eNOS pathways (70), whereas HMB primarily promotes muscle synthesis through mTOR with minimal effects on IMF (71). Within the Hippo pathway, Wnt proteins (*WNT9A*, *WNT10B*, *WNT6*, *WNT2B*) bind FZD receptors to recruit DVL and inhibit GSK-3β, stabilizing β-catenin for nuclear translocation (72). Nuclear β-catenin binds TCF/LEF to transcribe anti-apoptotic genes (Cyclin D1, c-Myc), promoting myocyte survival/proliferation (73). RES-HMB additive this mechanism—RES modulates fat deposition via PPARγ, while HMB activates mTOR-mediated protein synthesis, collectively optimizing IMF distribution for superior meat quality (74, 75). In Arachidonic acid metabolism, *PTGS2* converts arachidonic acid to PGH2, which is metabolized to PGG2 or *PTGD2* by *PTGDS* (76); alternatively, *PLA2G4* generates lecithin, while *ALOX12* oxidizes arachidonic acid to 12(S)-HPETE and 12(S)-HETE, regulating inflammation and signaling (77, 78). RES-HMB co-treatment promotes PGH2-to-*PTGD2* conversion while suppressing *ALOX12*/12(S)-HETE, reducing inflammatory lipolysis and enhancing IMF deposition to improve flavor and juiciness (79, 80). Monotherapies show limited effects: RES mildly inhibits lipolysis, and HMB primarily stimulates muscle growth, underscoring their synergistic efficacy (81, 82).

The lipid metabolome serves as the central driver regulating meat flavor formation (83). In agreement, nutritional interventions targeting lipid metabolism, such as protected amino acids and plant-derived bioactives, have been reported to modulate tissue fatty acid profiles and microbial metabolites, enhancing sensory attributes and shelf-life (84, 85). This study systematically elucidated the coordinated regulatory network of glycerophospholipid metabolism, Arachidonic acid metabolism, linoleic acid metabolism, alpha-linolenic acid metabolism, and ether lipid metabolism, along with their mechanistic impacts on meat flavor. In glycerophospholipid metabolism, LPC (20:0) and LPC (20:1)—lysophosphatidylcholine metabolites—directly influence membrane physical properties and biological functions (86). Meanwhile, certain phosphatidylcholines may generate glycerol-3-phosphate via hydrolysis, which is subsequently converted to 1-acyl-glycerone-3P via the glycerone-P intermediate under GNPAT/GAT catalysis, ultimately participating in ether lipid biosynthesis and affecting membrane stability and signaling (87, 88). RES-HMB co-treatment significantly modulates these processes: RES activates SIRT1 to promote saturated phospholipid conversion to ether lipids, enhancing membrane stability (89), while HMB suppresses mTOR to reduce unsaturated lysophospholipid [e.g., LPC (20:1)] generation, mitigating lipid peroxidation (90). Their synergy inhibits aldehyde formation while promoting ether lipid-derived flavor precursors, simultaneously improving oxidative stability and flavor quality (12, 53). In Arachidonic acid metabolism, CYP4A/CYP4F-generated 20-HETE is metabolized by CYP2U to arachidonate, entering Linoleic acid metabolism, notably, PC (38:5) may serve as a key substrate/product in this pathway (91, 92). CYP2-family enzymes catalyze EETs formation, which EPHX2 hydrolyzes to dihydroxyepoxyeicosadienoic acids and tetrahydrofurandiols, modulating vascular tone, inflammation, and lipid-mediated signaling (93). RES-HMB co-treatment inhibits CYP4A/CYP4F to reduce 20-HETE while activating sEH to convert EETs to DiHETEs, lowering proinflammatory factors (94, 95); HMB may indirectly affect Arachidonic acid metabolism through anti-inflammatory or metabolic regulatory pathways (96), their combination may improve meat flavor. For alpha-Linolenic acid metabolism, *PLA2G4*-hydrolyzed phosphatidylcholine [e.g., PC (21:2)] releases α-linolenic acid (ALA), which is metabolized via two routes: FADS2-mediated desaturation to stearidonic acid (SDA) (97–99), or hydroxylation to 17-hydroxy-linolenic acid and volicitin, involved in PUFA elongation and plant defense signaling (100). RES upregulates FADS2 to boost ALA → SDA conversion, increasing ω-3 PUFAs (101), while HMB inhibits lipoxygenase to reduce 17-hydroxy-linolenic acid (102, 103). Their synergy diminishes grassy off-flavors by reducing lipid oxidation products (e.g., hexanal) while enhancing fresh aroma via PUFA-derived flavor compounds (C6-C9 aldehydes/ketones) (54, 104).

This study revealed the interaction mechanisms between gene expression regulation and lipid metabolic networks through integrated transcriptomic and lipidomic analyses, elucidating the biosynthetic pathways of key metabolites influencing meat flavor and their molecular regulatory basis (83). The analysis demonstrated that both transcriptomic and lipidomic data were co-enriched in the arachidonic acid (AA) metabolism pathway, which showed a significant association with the linoleic acid (LA) metabolism pathway in the lipidome. Within the AA (20:4) and LA (18:2) metabolic networks, phospholipase *PLA2G4A* (cytosolic phospholipase A2α) played a central regulatory role by specifically recognizing and hydrolyzing phospholipids containing polyunsaturated fatty acids [e.g., PC (38:5)], releasing free AA (20:4) from their sn-2 position (105). Specifically, PC (38:5) (likely containing an AA chain at the sn-2 position) was catalyzed by *PLA2G4A* to generate lysophosphatidylcholine (LPC) and free AA. The liberated AA was subsequently metabolized through two key pathways: first, oxidation by *ALOX12* (12-lipoxygenase) to produce 12-HPETE, which could be further converted into anti-inflammatory lipoxin mediators (97, 106, 107); and second, conversion into PGD2 via *PTGDS* (prostaglandin D2 synthase), an important pro-inflammatory and immunomodulatory molecule involved in flavor formation (108). Notably, phospholipids such as LPC (20:0), LPC (20:1), PC (21:2), and PC (37:0) were also hydrolyzed by *PLA2G4A* but primarily participated in membrane phospholipid remodeling. These findings provide novel theoretical insights into the molecular mechanisms underlying meat flavor formation, suggesting that the *PLA2G4A*-AA-*ALOX12*/*PTGDS* axis may serve as a key metabolic hub regulating meat flavor quality. Comparable mechanisms of lipid metabolism modulation have been identified in other animal and plant systems through transcriptomic and metabolomic profiling, confirming the significance of integrated multi-omics approaches (109, 110).

## 5 Conclusion

Our study demonstrated that the H-RES-HMB group significantly reduced the intermuscular adipocyte area and diameter, increased cell density, and optimized fatty acid composition (decreasing SFAs while increasing MUFAs/PUFAs) as well as flavor compounds (ketones and esters). Transcriptomic analysis revealed that RES and HMB synergistically regulated the Calcium, Hippo, Estrogen, and Arachidonic acid pathways, promoting muscle cell proliferation, modulating fat deposition, and improving meat quality. Lipid metabolomics indicated that the *PLA2G4A*-AA-*ALOX12*/*PTGDS* axis hydrolyzed PC (38:5) to release AA, regulating the generation of flavor precursors, while LPC (20:0/20:1) and others participated in membrane remodeling, achieving a “reducing off-flavor and enhancing aroma” effect. Integrated analysis identified this axis as a key metabolic hub for flavor regulation.

## Data Availability

The datasets presented in this study can be found in online repositories. The names of the repository/repositories and accession number(s) are NCBI SRA (accession: PRJNA1205084) and OMIX (accession: OMIX011438).
