# Supplementary material for: Dietary resveratrol and β-Hydroxy-β-Methylbutyric acid enhance flavor and modulate intramuscular fat in Tibetan sheep: insights from transcriptomics and lipidomics
Source: Front Vet Sci. 2025 Sep 3;12:1634086. doi: 10.3389/fvets.2025.1634086 (PMC12440778; doi:10.3389/fvets.2025.1634086)
Supplement: Supplementary file 1 [file Data_Sheet_1.zip › Table S1/Table 1.docx]

| Sample | Clean reads | Total | Unmapped（%） | Unique Mapped（%） | Multiple Mapped（%） | Total Mapped  （%） |
| --- | --- | --- | --- | --- | --- | --- |
| H1 | 46186478 | 45968434 | 1998417  (4.35%) | 40889096  (88.95%) | 3080921  (6.70%) | 43970017  (95.65%) |
| H2 | 41292968 | 41096624 | 1820655  (4.43%) | 36561662  (88.97%) | 2714307  (6.60%) | 39275969  (95.57%) |
| H3 | 42468432 | 42259686 | 1853014  (4.38%) | 37594972  (88.96%) | 2811700  (6.65%) | 40406672  (95.62%) |
| H4 | 41255040 | 41063400 | 1719126  (4.19%) | 36605565  (89.14%) | 2738709  (6.67%) | 39344274  (95.81%) |
| H-RES-1 | 39168512 | 39054420 | 1754610  (4.49%) | 34928493  (89.44%) | 2371317  (6.07%) | 37299810  (95.51%) |
| H-RES-2 | 41624144 | 41516166 | 2020705  (4.87%) | 36957207  (89.02%) | 2538254  (6.11%) | 39495461  (95.13%) |
| H-RES-3 | 41093256 | 40976022 | 2046700  (4.99%) | 36355104  (88.72%) | 2574218  (6.28%) | 38929322  (95.01%) |
| H-RES-4 | 36046842 | 35932050 | 1669249  (4.65%) | 32001312  (89.06%) | 2261489  (6.29%) | 34262801  (95.35%) |
| H-HMB-1 | 36599588 | 36495602 | 1696398  (4.65%) | 32696078  (89.59%) | 2103126  (5.76%) | 34799204  (95.35%) |
| H-HMB-2 | 36962308 | 36873946 | 1762108  (4.78%) | 32999437  (89.49%) | 2112401  (5.73%) | 35111838  (95.22%) |
| H-HMB-3 | 40237516 | 40141854 | 2058936  (5.13%) | 35753359  (89.07%) | 2329559  (5.80%) | 38082918  (94.87%) |
| H-HMB-4 | 38097996 | 38005710 | 2048521  (5.39%) | 33721256  (88.73%) | 2235933  (5.88%) | 35957189  (94.61%) |
| H-RES-HMB-1 | 38848958 | 38751928 | 1725910  (4.45%) | 34956146  (90.20%) | 2069872  (5.34%) | 37026018  (95.55%) |
| H-RES-HMB-2 | 37003774 | 36900576 | 1716615  (4.65%) | 33200995  (89.97%) | 1982966  (5.37%) | 35183961  (95.35%) |
| H-RES-HMB-3 | 36756060 | 36660202 | 1689456  (4.61%) | 32980310  (89.96%) | 1990436  (5.43%) | 34970746  (95.39%) |
| H-RES-HMB-4 | 39619262 | 39477920 | 1948367  (4.94%) | 35360157  (89.57%) | 2169396  (5.50%) | 37529553  (95.06%) |
