# Supplementary material for: Dietary resveratrol and β-Hydroxy-β-Methylbutyric acid enhance flavor and modulate intramuscular fat in Tibetan sheep: insights from transcriptomics and lipidomics
Source: Front Vet Sci. 2025 Sep 3;12:1634086. doi: 10.3389/fvets.2025.1634086 (PMC12440778; doi:10.3389/fvets.2025.1634086)
Supplement: Supplementary file 1 [file Data_Sheet_1.zip › Table S1/Table 4.docx]

| Type/POS离子模式 | num | percentage(%) | Type/NEG离子模式 | num | percentage(%) |
| --- | --- | --- | --- | --- | --- |
| all | 2136 | 100 | all | 1402 | 100 |
| TG | 821 | 38.436 | PC | 229 | 16.334 |
| DG | 332 | 15.543 | PE | 175 | 12.482 |
| PE | 193 | 9.036 | Cer | 169 | 12.054 |
| PC | 161 | 7.537 | Hex1Cer | 153 | 10.913 |
| Hex1Cer | 154 | 7.210 | PS | 131 | 9.344 |
| Cer | 128 | 5.993 | CL | 114 | 8.131 |
| SM | 66 | 3.090 | PI | 55 | 3.923 |
| WE | 45 | 2.107 | SM | 51 | 3.638 |
| ZyE | 31 | 1.451 | Hex2Cer | 47 | 3.352 |
| LPC | 24 | 1.124 | PG | 42 | 2.996 |
